# CH_3_NH_3_Br solution as a novel platform for the selective fluorescence detection of Pb^2+^ ions

**DOI:** 10.1038/s41598-019-52431-y

**Published:** 2019-11-01

**Authors:** Jun Yan, Yuchun He, Yunlin Chen, Yongzhe Zhang, Hui Yan

**Affiliations:** 1 Institute of Applied Micro-Nano Materials, School of Science, Beijing Jiaotong University, Beijing, 100044 People’s Republic of China; 2 College of Materials Science and Engineering, Beijing University of Technology, Beijing, 100124 P.R. China

**Keywords:** Materials science, Nanoscale materials, Organic-inorganic nanostructures

## Abstract

The development of a simple fluorescent sensor for detecting the Pb^2+^ heavy metal is fundamentally important. The CH_3_NH_3_PbBr_3_ perovskite material exhibits excellent photoluminescence properties that are related to Pb^2+^. Based on the effects of Pb^2+^ on the luminescent properties of CH_3_NH_3_PbBr_3_, we design a novel platform for the selective fluorescence detection of Pb^2+^ ions. Herein, we use a CH_3_NH_3_Br solution at a high concentration as the fluorescent probe. Incorporation of PbBr_2_ into the CH_3_NH_3_Br solution results in a rapid chemical reaction to form CH_3_NH_3_PbBr_3_. Hence, the nonfluorescent CH_3_NH_3_Br material displays a sensitive and selective luminescent response to Pb^2+^ under UV light illumination. Moreover, the reaction between CH_3_NH_3_Br and PbBr_2_ could transform Pb^2+^ into CH_3_NH_3_PbBr_3_, and therefore, CH_3_NH_3_Br may also be used to extract Pb^2+^ from liquid waste in recycling applications.

## Introduction

In the past several decades, the control of heavy metal pollution has been the focal point of environmental protection efforts^1–6^. Development of simple and selective sensors is critical for detection of heavy metals. Several methods such as atomic absorption spectrometry (AAS)^7^, inductively coupled plasma-mass spectrometry (ICP-MS)^8^, inductively coupled plasma atomic-emission spectroscopy (ICP-AES)^9^, electrochemical methods^10^, and fluorescent techniques^11^ have been devised to detect heavy metals. Compared with other methods, the fluorescence-based methods display many advantages such as low cost, high sensitivity, rapid detection, and ease of use^12^. As fluorescent materials, lead halide perovskite CH_3_NH_3_PbBr_3_ (MAPbBr_3_) and CsPbBr_3_ show excellent luminescent properties including bright photoluminescence (PL), high PL quantum yields (PLQY), and narrow bandwidth^13–15^. Compared with MAPbBr_3_, the PL emission peaks of MAPbI_3_ and MAPbCl_3_ are red and blue light, respectively, which can’t be excited by a UV lamp. Due to these advantages, the MAPbBr_3_ and CsPbBr_3_ have been used in light-emitting diodes (LED)^16–22^ and fluorescence sensors or detectors^12,23^. Chinnadurai *et al*.^24^ reported that fluorescent MAPbBr_3_ nanoparticles can be used as an excellent sensor for the detection of 2, 4, 6-trinitrophenol (TNP). Liu *et al*.^25^ used CsPbBr_3_ perovskite quantum dots as photoluminescent probe for selective detection of Cu^2+^. Zhang *et al*.^12^ encapsulated MAPbBr_3_ perovskite quantum dots in MOF-5 matrix as a stable fluorescent probe for the detection of Al^3+^, Bi^3+^, Co^2+^, Cu^2+^, Fe^3+^, and Cd^2+^. The detection mechanisms of the perovskite fluorescent sensor is mostly related to luminescence-quenching mechanisms. The introduction of metal ions in perovskite solutions will quench the PL performance of perovskite materials. However, the excellent PL properties of MAPbBr_3_ and CsPbBr_3_ are due to the Pb^2+^ ion. The high toxicity of Pb^2+^ is a considerable concern for the future applications of the lead halide perovskite fluorescent probe. In this work, we report on the use of MABr solution for the selective and sensitive detection of Pb^2+^. The MABr solution detects the Pb^2+^ due to the luminescence enhancing effect which is different from the quenching mechanisms of lead halide perovskite fluorescent probes.

## Experimental Section

All materials were purchased from Xi’an Polymer Light Technology Corp (China). The MABr solution was prepared by dissolving 0.8 mmol MABr in 1 ml N, N-dimethylformamide (DMF). To detect the Pb^2+^ concentration, different amounts of PbBr_2_, PbI_2_ and PbCl_2_ powders were added into MABr solutions to form the MABr@PbBr_2_, MABr@PbI_2_and MABr@PbCl_2_ precursor solutions, respectively. After stirring the precursor solutions at room temperature for 30 min, the MABr@PbBr_2_, MABr@PbI_2_ and MABr@PbCl_2_ solutions were transformed into MABr@MAPbBr_3_, MABr@MAPbBr_3−x_I_x_ and MABr@MAPbBr_3−x_Cl_x_solutions that are transparent liquids under room light. The photoluminescence (PL) emission spectra of the MABr@MAPbBr_3_, MABr@MAPbBr_3−x_I_x_ and MABr@MAPbBr_3−x_Cl_x_ solutions were measured by a photoluminescence system in the reflection mode. The time-resolved PL spectra of MABr@MAPbBr_3_ solution were measured by an FLS980 time-resolved fluorescence spectrometer (Edinburgh Instrument). To analyze the structures of these solutions, MABr@MAPbBr_3_ solutions were dropped on the glass substrate and then heated at 100 °C for 30 min in order to evaporate the DMF solvents. After the heat treatment, the precipitates of MABr@MAPbBr_3_ solutions were formed on the substrate. For all of the samples on the substrates, X-ray diffraction (XRD) and scanning electron microscopy (SEM) measurements were carried out to analyze the crystal structure and morphology of the precipitate, respectively. Dynamic light scattering measurement (DLS) was conducted to analyze the size distributions of the particles in MABr and MABr@MAPbBr_3_ solutions.

## Results and Discussion

**Figure 1:**
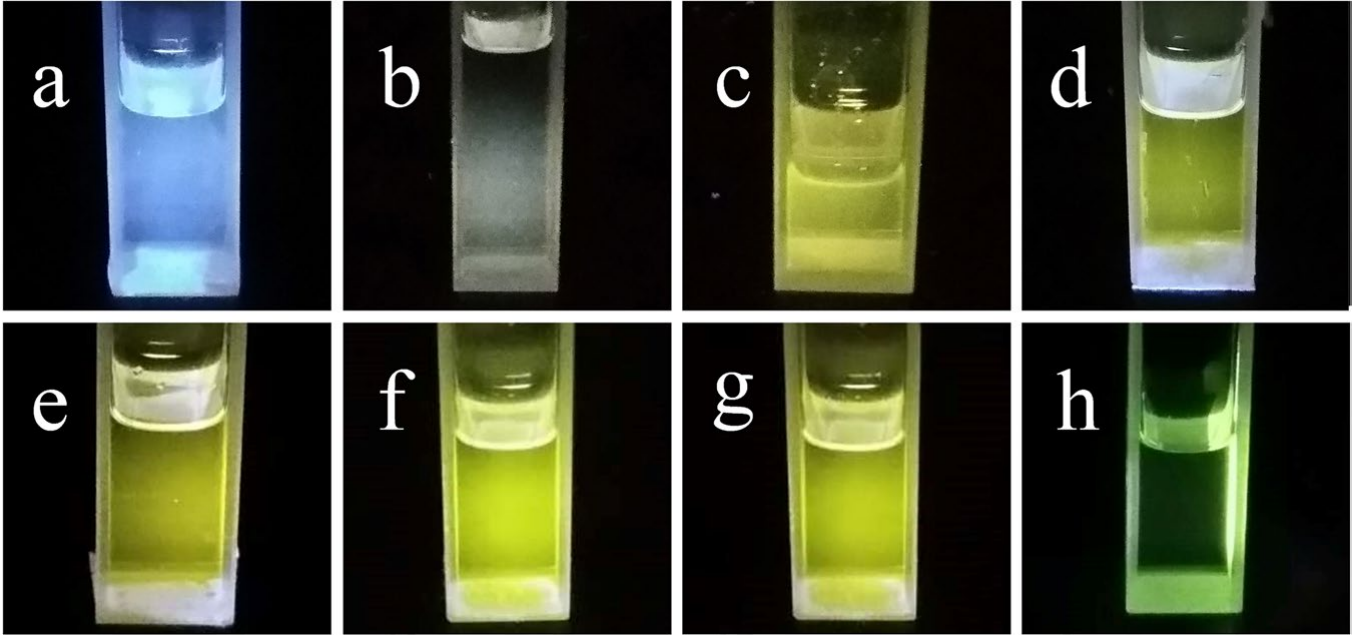
Photographs of MABr@MAPbBr_3_ solutions under illumination by a 365 nm UV lamp; Pb^2+^ concentration: (**a**) 0 M (**b**) 1.6 × 10^−3^ M (**c**) 3.1 × 10^−3^ M (**d**) 6.2 × 10^−3^ M **(e**) 2.5 × 10^−2^ M (**f**) 5 × 10^−2^ M (**g**) 1 × 10^−1^ M (**h**) 2 × 10^−1^ M.

**Figure 2:**
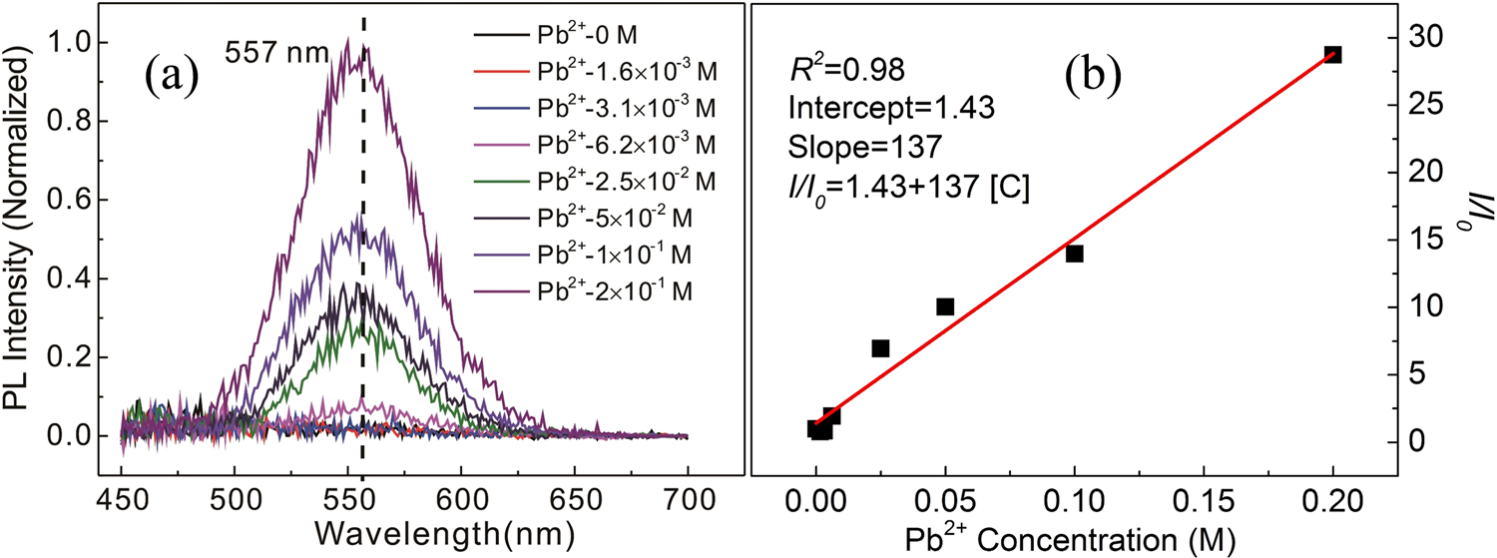
(**a**) PL emission spectra of MABr@MAPbBr_3_ solutions for different Pb^2+^ concentrations for an excitation wavelength of 400 nm. (**b**) Fitting curve for the PL intensity of the MABr@MAPbBr_3_ solutions as a function of Pb^2+^ concentration.

Figure 1 shows the photographs of MABr@MAPbBr_3_ solutions under 365 nm UV lamp in a darkroom. As shown in Fig. 1(a–h), the solutions consisted of 0.8 M MABr and different amounts of PbBr_2_ (0–2 × 10^−1^ M). All of the solutions were transparent under ambient light. The MABr solution without PbBr_2_ only reflects the purple color of the UV light under the UV lamp illumination, as shown in Fig. 1(a), indicating that the MABr solution is nonfluorescent under the UV lamp illumination. Introduction of a small amount of PbBr_2_ to the MABr solution leads to the formation of the MABr@MAPbBr_3_ solution, and the MABr@MAPbBr_3_ solution emits very pale yellow color under UV light illumination. As the Pb^2+^ concentration of the MABr@MAPbBr_3_ solutions increased from 1.6 × 10^−3^ to 2 × 10^−1^ M, the emission colors of these solutions changed quickly from pale yellow to bright green, as shown in Fig. 1(b–h). The dependence of the photoluminescence (PL) of the MABr@MAPbBr_3_ solutions on Pb^2+^ concentration is displayed in Fig. 2(a). All of the solutions were measured at room temperature with an excitation wavelength of 400 nm. The MABr solution does not show any florescence signal and the MABr@MAPbBr_3_ solutions exhibit a green emission peak centered at 557 nm. However, the green emission peaks of the solutions display a large full-width-at-half-maximum (FWFM). For the MABr@MAPbBr_3_ solution with 2 × 10^−1^ M Pb^2+^, the FWFM of emission peak is 60 nm, which is larger than that of the MABrPb_3_ thin film and powder^26–30^. Hence, a yellow green color emission is observed from the MABr@MAPbBr_3_ solutions under the 365 nm UV lamp illumination in a darkroom (Fig. 1). The PL intensity of the MABr solution was significantly increased by the addition of Pb^2+^ ion in a concentration-dependent manner (Pb^2+^ concentration ranging from 0 to 2 × 10^−1^ M). The influence of MABr concentration on the sensitivity of Pb^2+^ detection was studied (Fig. S1). If the Pb^2+^ concentration is greater than 1 × 10^−1^ M, then for the same Pb^2+^ concentration, the PL intensity of the 0.8 M MABr solution is nearly the same as that of the 0.4 M MABr solution (Fig. S1). However, upon a further decrease in the Pb^2+^ concentration, the PL intensity of the 0.4 M MABr solution is much smaller that of the 0.8 M MABr solution. Hence, increasing the concentration of MABr will enhance the sensitivity for detection of Pb^2+^ ions. However, if the MABr concentration is larger than 0.8 M, the MABr powder is insoluble in the DMF solution at room temperature. Therefore, we choose the 0.8 M MABr solution as the fluorescent probe for the detection of Pb^2+^ ions.

**Figure 3:**
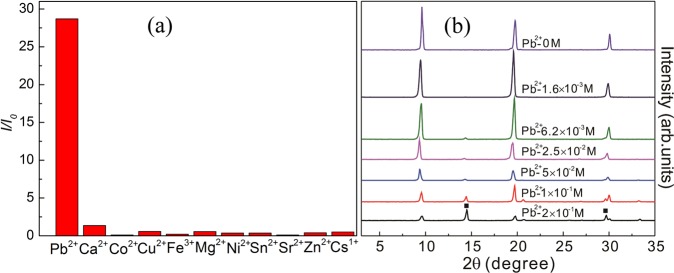
(**a**) PL response of the MABr solution (0.8 M) to different metal ions. (**b**) X-ray diffraction patterns of MABr@MAPbBr_3_ solutions with different Pb^2+^ concentrations.

To clarify the general applicability of MABr for Pb^2+^ detection, we replace the PbBr_2_ powders with PbI_2_ and PbCl_2_ powders to form the MABr@PbI_2_ and MABr@PbCl_2_ precursor solutions, respectively. Figure S2 shows the PL emission spectra of MABr@PbI_2_ and MABr@PbCl_2_ solutions for different Pb^2+^ concentrations. Compared with MABr solutions, both MABr@PbI_2_ and MABr@PbCl_2_ solutions display a green emission peak under UV light illumination. For MABr@PbI_2_ or MABr@PbCl_2_ solutions, with I^−^ or Cl^−^ concentrations increase, the center of the PL emission peak gradually changed. Based on the PL measurement of MABr, MABr@PbBr_2_, MABr@PbI_2_ and MABr@PbCl_2_ solutions, we can get the conclusion that MABr solutions exhibit luminescent response to Pb^2+^ ions. To obtain the quantitative relationship, we plotted the PL intensity of the solutions as a function of Pb^2+^ concentration (Fig. 2(b)). The relationship can be described by the following equation:

$$I/{I}_{0}=A+K[{\rm{C}}]$$

where *I* and *I*_0_ are the PL intensities of the solution in the presence and absence of Pb^2+^ ions, respectively. *A* and *K* (1/M) are the intercept and sensitivity (slope), respectively, and [C] (M) represents the Pb^2+^ concentration. The *I*/*I*_0_ − Pb^2+^ concentration curve can be fitted to *I*/*I*_0_ ± Δ (*I*/*I*_0_) = 1.43 ± 0.59 + (137.00 ± 7.22) [C], with the correlation coefficient R^2^ of 0.98, as shown in Fig. 2(b). The Δ(*I*/*I*_0_), 0.59 and 7.22 are the standard error of *I*/*I*_0_, *A* and *K* respectively. It was reported that perovskite fluorescent materials for the selective detection of metal ions or 2, 4, 6-trinitrophenol (TNP) are based on the quenching mechanism and the Stern-Volmer relationship^31^. However, the PL emission intensity of the MABr solution was enhanced with the addition of Pb^2+^, which is different from the quenching mechanism. To evaluate the selective detection ability of the MABr solution for Pb^2+^ ions, the PL response of the MABr solution to different metal ions was explored, as shown in Fig. 3(a). The PL intensity (*I*) of the MABr solutions (0.8 M) after the addition of different metal ions in the same concentration (2 × 10^−1^ M), including Pb^2+^, Ga^2+^, Co^2+^, Cu^2+^, Fe^3+^, Mg^2+^, Ni^2+^, Sn^2+^, Sr^2+^, and Zn^2+^, Cs^1+^ were measured. Analysis of the PL intensity ratios *I/I*_0_ (*I*_0_ is the PL intensity of the MABr solution without the metal ions) of the MABr solutions with different ions showed that only Pb^2+^ gave rise to a clear PL effect for the MABr solutions, while other cations exhibit almost no PL behavior for an excitation wavelength of 400 nm or under illumination by a 365 nm UV lamp.

In order to get a better insight into the role of MABr on selective fluorescence detection of Pb^2+^ ions, the effect of interference by other metal ions were studied. Some equimolar mixtures of PbBr_2_ (0.1 M) along with XBr (X = different metal ions) were added to the MABr solutions. The PL response of the MABr solution to equimolar mixtures of Pb^2+^ with different metal ions is shown in Fig. S3. For transparent solutions (Pb^2+^@Ca^2+^, Pb^2+^@Mg^2+^, Pb^2+^@Sn^2+^, Pb^2+^@Sr^2+^, Pb^2+^@Zn^2+^, Pb^2+^@Cs^+^), the presence of interfering ions has little impact on the luminescent response of the MABr solution to Pb^2+^. For semitransparent solutions Pb^2+^@Co^2+^ and Pb^2+^@Ni^2+^, their PL intensities are greatly lower than that of MABr@PbBr_2_ solutions. However, compared with MABr, the MABr@PbBr_2_@CoBr_2_ and MABr@PbBr_2_@NiBr_2_ solutions still exhibit a green PL emission peak. The MABr@PbBr_2_@CoBr_2_ solution emits green color under UV light illumination as shown in Fig. S4. Hence, the MABr can be used to selectively detect the Pb^2+^ in the presence of Ca^2+^, Mg^2+^, Sn^2+^, Sr^2+^, Zn^2+^, Cs^+^, Co^2+^, Ni^2+^. For opaque solutions Pb^2+^@Cu^2+^ and Pb^2+^@Fe^3+^, the MABr@PbBr_2_@CuBr_2_ and MABr@PbBr_2_@FeBr_3_ solutions do not show any florescence signal. Researchers also reported that Cu^2+^ leaded to dramatic quenching of the PL of perovskite materials^11,12,25^. Therefore, the MABr can’t detect the Pb^2+^ in Pb^2+^@Cu^2+^ or Pb^2+^@Fe^3+^ solutions.

**Figure 4:**
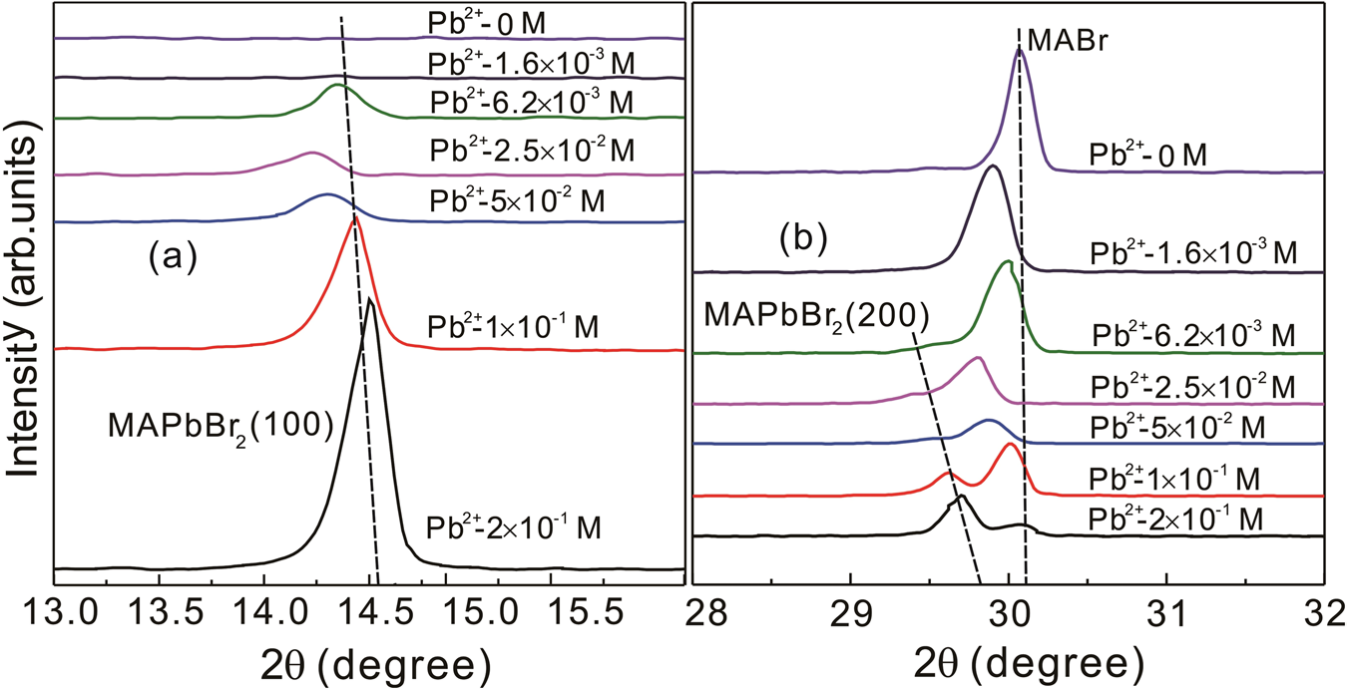
XRD peaks of MAPbBr_3_ in MABr@MAPbBr_3_ solutions with different Pb^2+^ concentrations (**a**) (100) and (**b**) (200) peak.

To explain the significant selective luminescent response of MABr solutions to Pb^2+^, the structure of MABr@MAPbBr_3_ solutions with different Pb^2+^ concentrations should be elucidated. For all of the solutions, the DMF solvents were evaporated on the glass substrates, and then we obtained MABr@MAPbBr_3_ films that were analyzed by XRD. Figure 3(b) shows the XRD patterns of these films with different Pb^2+^ concentrations from top to bottom at room temperature. The XRD patterns of the MABr powder and MABr thin films were measured (Fig. S5). For the MABr solutions without PbBr_2_ (top spectrum in Fig. 3(b)), all of the diffraction peaks are the characteristic peaks of MABr. As Pb^2+^ concentration increases, the MABr peaks intensities gradually decrease and new diffraction peaks of MAPbBr_3_ indicated by squares appear in the XRD patterns presented in Fig. 3(b). The evolution of MAPbBr_3_ peaks with Pb^2+^ concentration is shown in Fig. 4(a,b). The XRD results (Fig. 4) show that the two peaks’ intensities gradually increase with increasing Pb^2+^ concentration, corresponding to the increasing crystalline characteristics of MAPbBr_3_ with a preferential orientation in the (100) and (200) directions. Our previous study also found that MAPbBr_3_ thin films prepared with high MABr concentration exhibit partial preferential orientation along the (100) and (200) directions^28,29^. The (100) and (200) peaks shift toward larger angles with increasing Pb^2+^ concentration, indicating that the lattice constant of MAPbBr_3_ is decreasing. Due to the preferential orientation, we calculated the lattice parameters of MAPbBr_3_ from the (100) and (200) diffraction peaks. For the film with 2 × 10^−1^ M Pb^2+^, the lattice constant of MAPbBr_3_ is 6.10 Å which is larger than the previously reported value^28,29^. However, our research indicated that when Pb^2+^ concentration increased to 1 × 10^−1^ M, the lattice constant of MAPbBr_3_ was 5.93 Å which is closer to the values reported by other researchers^26,27^. To further confirm the MAPbBr_3_ phase in MABr@MAPbBr_3_ films, we studied the morphology of these films with SEM measurements.

**Figure 5:**
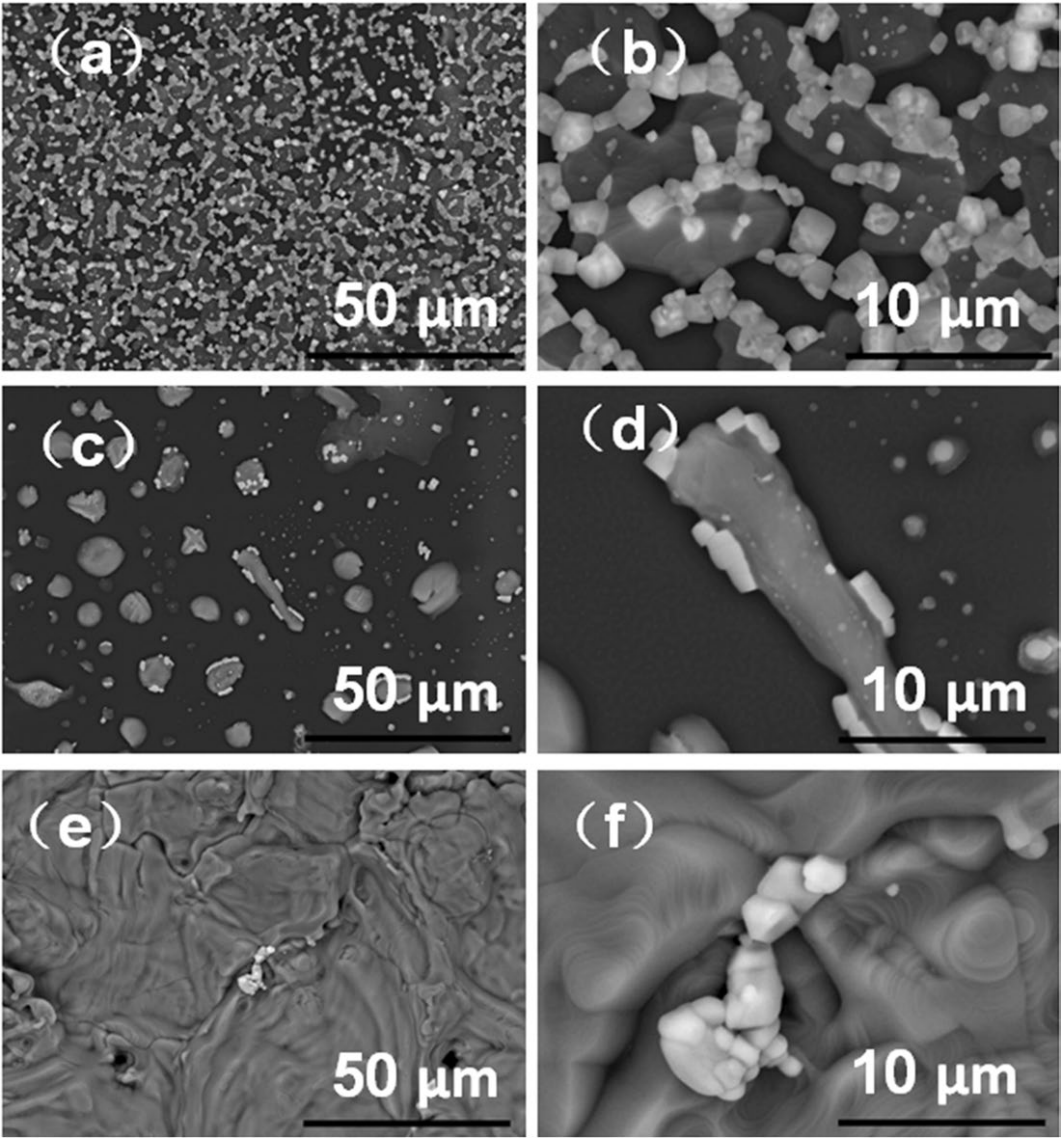
Low-magnification and high-magnification SEM images of the MABr@MAPbBr_3_ solutions with different Pb^2+^ concentrations; Pb^2+^− 2 × 10^−1^ M (**a,b**), Pb^2+^− 5 × 10^−2^ M (**c,d**), Pb^2+^− 1.6 × 10^−3^ M (**e,f**).

Figure 5 shows the morphologies of the films prepared with MABr@MAPbBr_3_ solutions with different Pb^2+^ concentrations. An examination of Fig. 5(a,b) shows that the surface exhibits two different nonuniform aggregation morphologies. The first type of aggregation shows a shapeless morphology which is characteristic of the MABr organic compound. The other kind of aggregation is composed of cubic-shaped crystals, which is the crystalline morphology of MAPbBr_3_. The SEM results are consistent with the XRD analyses that indicate that this film is composed of MABr and MAPbBr_3_ phases as shown in Figs 3(b) and 4. When the PbBr_2_ concentration in the precursor solution is reduced, the number of MAPbBr_3_ crystals formed in the films decreased, as shown in Fig. 5(c,d). For the solution with 1.6 × 10^−3^ M Pb^2+^, the morphology of the film displays highly dense MABr aggregation with high coverage and only a small amount of MAPbBr_3_ crystals are observed on top of the MABr as shown in Fig. 5(e,f). We did not find any phases other than MABr and MAPbBr_3_ in the XRD patterns and SEM images of the films prepared by evaporating the DMF solvents from the MABr@MAPbBr_3_ solutions. To further exploring the interactions between MABr and PbBr_2_, the size distributions of the particles formed in MABr and MABr@MAPbBr_3_ solutions were measured by Dynamic Light Scattering (DLS), as shown in Fig. S6. The high MABr concentration (0.8 M) tends to form a gel-like solution and its hydrodynamic particle diameter is 650.1 nm, indicating the formation of MA^+^ organic aggregates. Adding PbBr_2_ in MABr solution increases the particles size. With Pb^2+^ concentration increased to 0.2 M, the diameter of the particles in MABr@MAPbBr_3_ solutions increased from 650.1 to 783.5 nm. The added PbBr_2_ might quickly react with Br^−^ and MA^+^ to generate PbBr_6_ octahedron inorganic frame and then self-assemble into MAPbBr_3_ perovskite lattice. Adding PbBr_2_ in MABr solutions would provide more nucleating sites and growth spaces, resulting in the formation of bigger aggregates in MABr@MAPbBr_3_ solutions. Hence, addition of a small amount of PbBr_2_ to a high MABr concentration solution and stirring of this mixture could lead to a rapid chemical reaction to form MAPbBr_3_.

The significant luminescent response of the MABr solution to Pb^2+^ arises from the outstanding photoluminescence properties of MAPbBr_3_. The detection limit is an important indicator of the fluorescence detector performance. As the PbBr_2_ concentration was reduced to 1.6 × 10^−3^ M, the PL emission peak and diffraction peaks associated with the MAPbB_3_ can no longer be observed in the PL emission spectra (Fig. 2) and XRD patterns (Figs 3(b) and 4), respectively. When the PbBr_2_ concentration is lower than 1.6 × 10^−3^ M, only the diffraction peak of MABr could be clearly observed in the XRD pattern. However, the MABr solution with 1.6 × 10^−3^ M PbBr_2_ shows a pale yellow color under UV light illumination and we can still find the MAPbBr_3_ crystals in SEM images, unlike for the MABr solution without PbBr_2_. Therefore, the detection limit of the 0.8 M MABr solution for Pb^2+^ is at least as low as 1.6 × 10^−3^ M. Compared with other methods^7–10^ whose detection limits for Pb^2+^ are μM, the sensitivity of the MABr is not high. However, the MABr fluorescent sensor also displays many advantages such as low cost, rapid detection and ease of use. For the MABr fluorescent sensor, to selectively detect the Pb^2+^ from other metal ions, we only need a UV lamp equipment which is very cheap and easy to use. Moreover, the high MABr concentration solutions can quickly react with Pb^2+^ to form MAPbBr_3_, which can be used to extract Pb^2+^ from liquid waste in recycling applications. Other heavy metal detectors can’t extract Pb^2+^ from liquid waste. We also justify the luminescent response of MABr to Pb^2+^ on paper strips, as shown in Fig. S7. The letters “BJTU” were written with PbBr_2_ solution (0.1 M) on paper strips. The “BJTU” are invisible on paper strips under ambient light. However, after loading of MABr solutions (0.8 M) on these paper strips, the “BJTU” show bright green emission pattern under UV light illumination.

**Figure 6:**
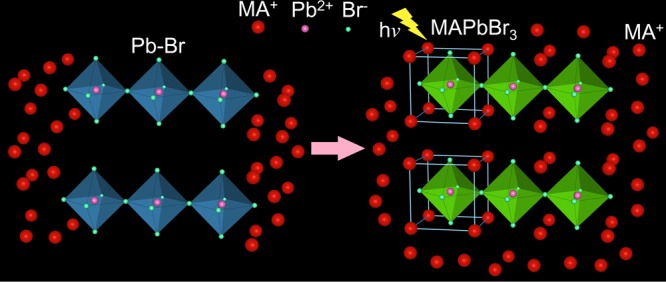
Schematic representation for the luminescent response of the MABr solution to Pb^2+^.

Based on these results, the fluorescence sensing mechanism can be schematically represented as shown in Fig. 6. To obtain excellent performance in a photovoltaic device, it is necessary to lower the rate of the chemical reaction between MABr and PbBr_2_ to form uniform MAPbBr_3_ films with good surface coverage^32–34^. However, for fluorescence sensors or detectors with a short response time, we seek to make MABr react with PbBr_2_ to form MAPbBr_3_ as quickly as possible. Excess MABr contributes to speeding up the transformation from PbBr_2_ to MAPbBr_3_. On the other hand, a MABr-rich environment gives rise to MABr residue that encompasses the MAPbBr_3_ crystal after the reaction. Thus, the use of excess MABr leads to the formation of a high amount of defects. The recombination lifetimes of the MABr@MAPbBr_3_ solution with 2 × 10^−1^ M Pb^2+^ is only 1.33 ns (Fig. S8). For perovskite materials, the recombination lifetime is related to crystallite dimension, the larger crystallites (e.g. single crystal) present longer photoluminescence lifetime. However, in our research, the DLS and SEM studies indicate that the MABr@MAPbBr_3_ solutions display big aggregates (783.5 nm) which consist of organic aggregates and MAPbBr_3_ crystals. Because of the organic aggregates and numerous defects, the MABr@MAPbBr_3_ solutions display a short-lived PL lifetime. In fact, the spin-coating method could evaporate some of the used MABr. We used a MABr@MAPbBr_3_ solution with 2 × 10^−1^ M Pb^2+^ as the precursor solution and spin-coated it on the glass substrate to obtain the MABr@MAPbBr_3_ thin film. The SEM images exhibit that the number of MABr residues of the MABr@MAPbBr_3_ thin film prepared by the spin-coating method is smaller than that of the MABr@MAPbBr_3_ film prepared by evaporating the DMF solutions (Figs 5(a,b) and S9). For MAPbBr_3_ films, a previous study also indicated that the introduction of Cl was conducive to the evaporation of the excess MABr during the spin-coating process^32–34^. If the MABr in MABr@MAPbBr_3_ solutions can be removed completely, we can obtain MAPbBr_3_ material with excellent photovoltaic performance. Therefore, the MABr solution not only can be used to detect the Pb^2+^ heavy metal but also may extract the Pb^2+^ from the liquid waste for reuse.

## Conclusion

In summary, our research indicates that MABr can be used as a new platform for selective fluorescence detection of Pb^2+^ ions. The incorporation of PbBr_2_ into a MABr solution formed MAPbBr_3_@MABr solutions that exhibit significant luminescent responses under UV light illumination. The significant color changes of the MABr solutions before and after the addition of PbBr_2_ under UV lamp illumination can be observed by the naked eye. The PL intensity of the MABr sensor increases with increasing Pb^2+^ concentration, exhibiting a linear relationship. The fluorescence sensing mechanism of MABr for Pb^2+^ is due to the excellent PL performance of MAPbBr_3_ in MAPbBr_3_@MABr solutions. Some MABr in MAPbBr_3_@MABr solutions can be evaporated by the spin-coating method, enabling the extraction of Pb^2+^ from the liquid waste for recycling use. These findings may contribute to the development of new applications for luminescent perovskite materials.

## Supplementary information

Supplementary Information

## Data Availability

The datasets analysed during the current study are available from the corresponding author on reasonable request.
