# Supplementary material for: CH3NH3Br solution as a novel platform for the selective fluorescence detection of Pb2+ ions
Source: Sci Rep. 2019 Nov 1;9:15840. doi: 10.1038/s41598-019-52431-y (PMC6825161; doi:10.1038/s41598-019-52431-y)
Supplement: Supplementary file 1 — Supplementary Information [file 41598_2019_52431_MOESM1_ESM.docx]

Supporting Information

**CH_3_NH_3_Br solution as a novel platform for the selective fluorescence** **detection of Pb^2+^ ions**

Jun Yan^1^, Yuchun He^1^, Yunlin Chen^1,*^, Yongzhe Zhang^2,*^, Hui, Yan^2^

^1^Institute of Applied Micro-Nano Materials, School of Science, Beijing Jiaotong University, Beijing 100044, People’s Republic of China

^2^College of Materials Science and Engineering, Beijing University of Technology, Beijing, 100124 P. R. China

*Correspondence and requests for materials should be addressed to Y. C. (email: ylchen@bjtu.edu.cn) or Y. Z. (email: yzzhang@bjut.edu.cn)


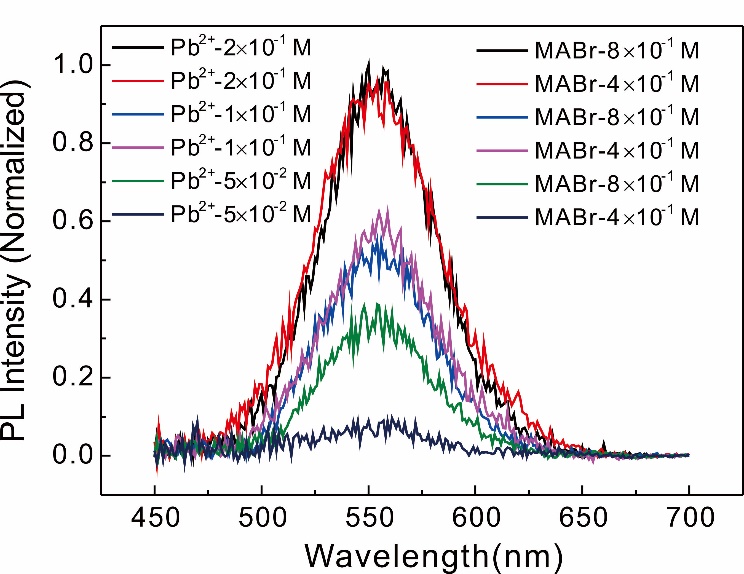


Figure S1. PL emission spectra of the MABr (0.4, 0.8 M) @MAPbBr_3_ solutions for different Pb^2+^ concentrations for an excitation wavelength of 400 nm.


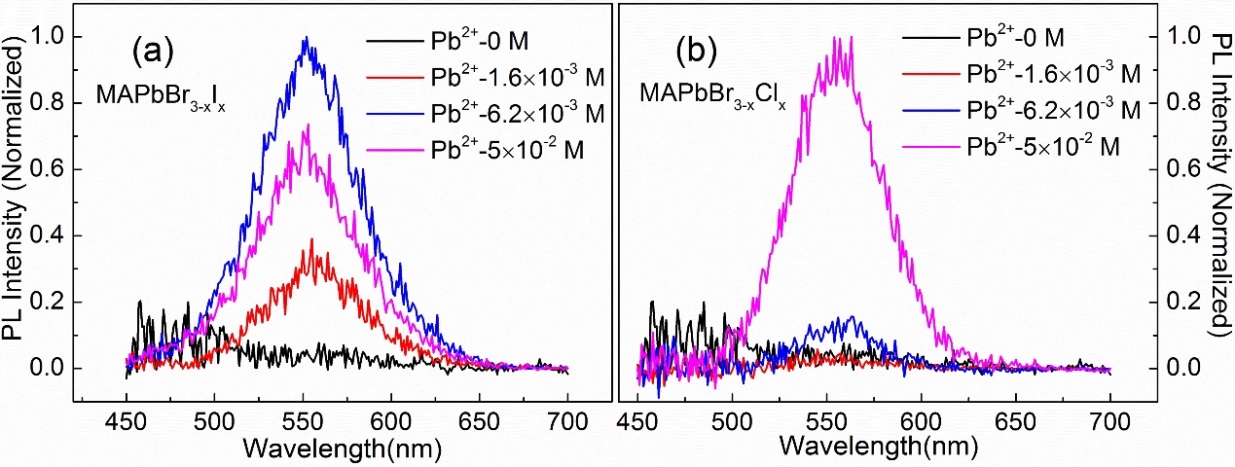


Figure S2. (a) PL emission spectra of (a) MABr (0.8 M)@PbI_2_ solutions and (b) MABr (0.8 M)@PbCl_2_ solutions for different Pb^2+^ concentrations for an excitation wavelength of 400 nm.


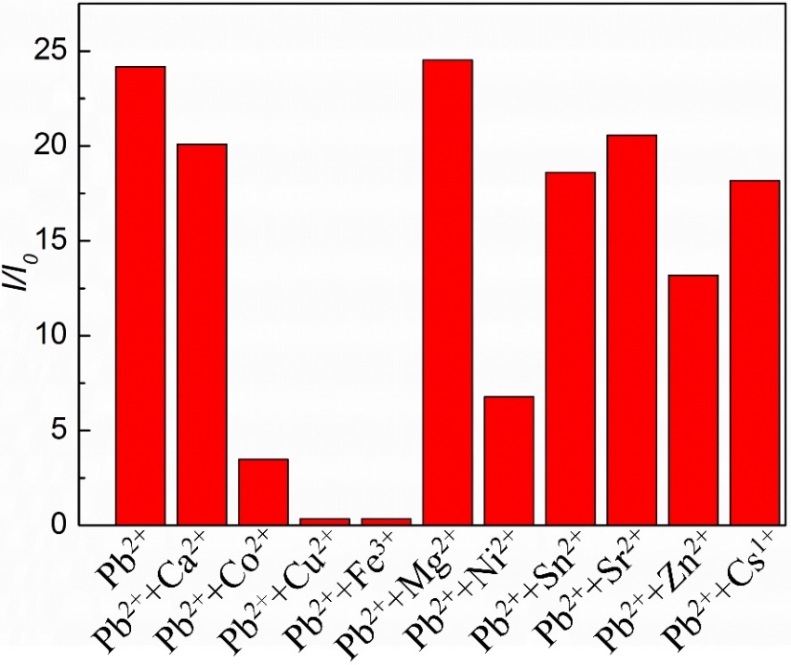


Figure S3. PL response of the MABr solution (0.8 M) to equimolar mixtures of Pb^2+^ (0.1 M) with different metal ions. The excitation wavelength is 400 nm.


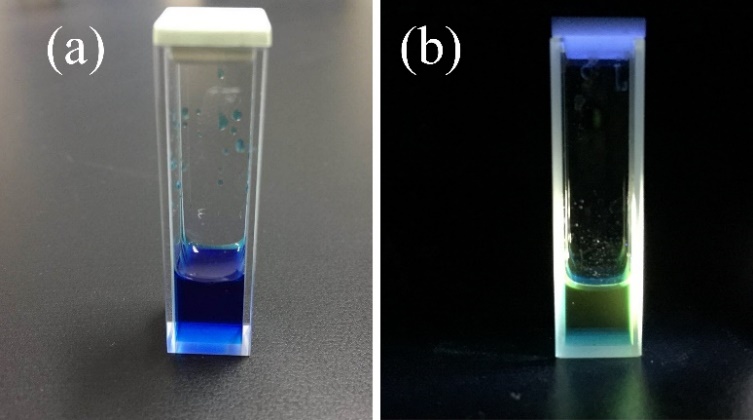


Figure S4. Photographs of MABr (0.8 M) mixed with PbBr_2_ (0.1 M) and CoBr_2_ solutions (0.1 M) under illumination by (a) ambient light (b) a 365 nm UV lamp.


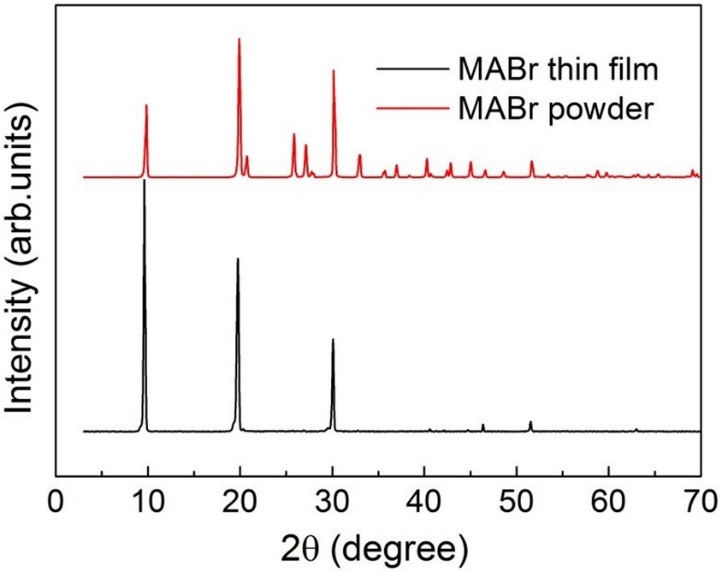


Figure S5. XRD patterns of MABr powder (top line) and MABr thin films (bottom line)


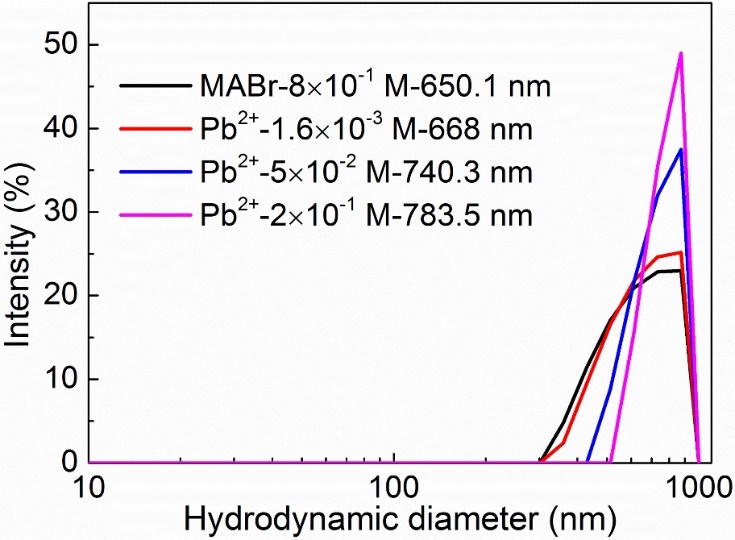


Figure S6. Size distributions of the particles formed in MABr and MABr@MAPbBr_3_ solutions with different Pb^2+^ concentrations.


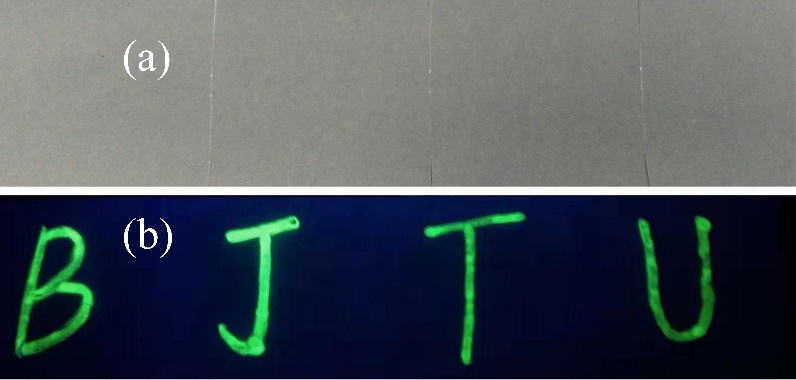


Figure S7. Digital images of the letters BJTU written with PbBr_2_ solution on a paper after MABr loading under (a) ambient light (b) a 365 nm UV lamp.


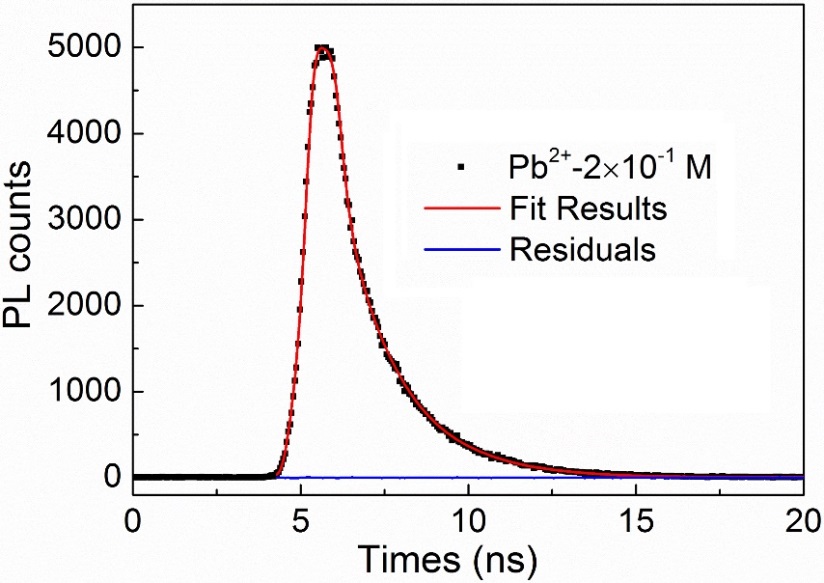


Figure S8. Time-resolved PL decay detected at the peak wavelength of emission for the MABr@MAPbBr_3_ solution with 2×10^-1^ M Pb^2+^.

Figure S8 shows PL decay curve of MABr@MAPbBr_3_ solution with 2×10^-1^ M Pb^2+^ at the emission peak wavelength. The PL decay curve was fitted by a biexponential function of time F(t)

$F(t)=\sum a_{i}e^{-t/\tau_{i}}$ (1)

where$a_{i}$ is a prefactor and $\tau_{i}$ is the time constant. The average recombination lifetimes ($\tau_{ave}$) of the solution was calculated by the following equation:

$\tau_{ave}=\sum a_{i}\tau_{i}^{2}/\sum a_{i}\tau_{i}$ (2)

The recombination lifetimes of the MABr@MAPbBr_3_ solution with 2×10^-1^ M Pb^2+^ is 1.33 ns.


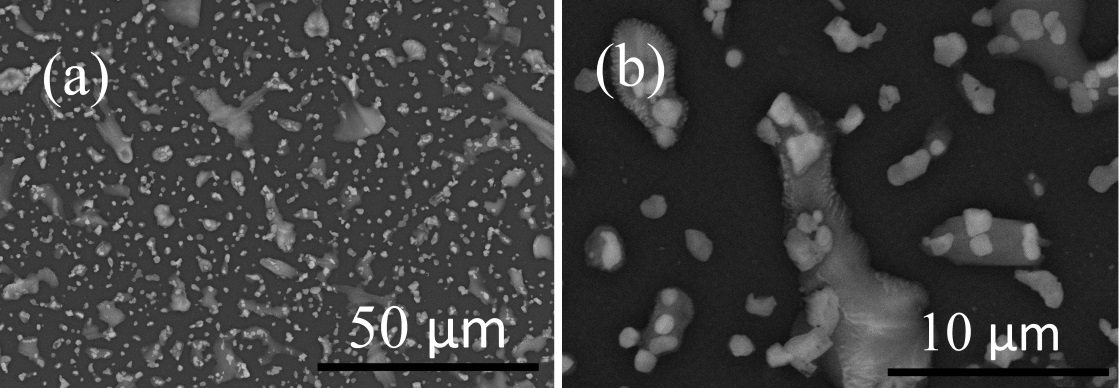


Figure S9. (a) Low-magnification and (b) high-magnification SEM images of the films prepared by spin coating the MABr@MAPbBr_3_ solutions with 2×10^-1^ M Pb^2+^ concentration.
